# Ectatic Coronary Arteries: Diversity at Its Finest

**DOI:** 10.7759/cureus.38381

**Published:** 2023-05-01

**Authors:** Najlaa Belharty, Oumaima Fertat, Tanae El Ghali, Fatine Tabti, Fatima Azzahra Benmessaoud, Latifa Oukerraj, Mohamed Cherti

**Affiliations:** 1 Department of Cardiology B, Ibn Sina Hospital, Mohammed V University, Rabat, MAR

**Keywords:** coronary slow flow, myocardial bridging, ectatic coronary vessel, coronary angiography, coronary ectasia, coronary artery

## Abstract

Coronary artery ectasia (CAE) is an entity causing inappropriate dilatation of the coronary tree, that is angiographically defined, albeit arbitrarily, by the diameter of the ectatic segment being more than 1.5 times larger in comparison with an adjacent healthy reference segment. Although the causative mechanisms are poorly understood, atherosclerosis is greatly implicated in the causation of CAE. Clinical, angiographic, and therapeutic features have been puzzling clinicians.

We illustrate three different angiographic subsets, co-existing with myocardial bridge/coronary slow flow and diversely presenting as asymptomatic, pauci, and frankly symptomatic with stable and acute coronary syndrome.

These cases illuminate the diversity of CAE's clinical and angiographic presentations and pathologic progression, shedding light on this medical condition and its implications.

## Introduction

Coronary artery ectasia (CAE) is an infrequent condition characterized by the excessive diffuse dilatation of the coronary arteries by 1.5 times in diameter compared to adjacent healthy coronary arteries [1]. Its prevalence in angiographic series varies between 0.2% and 10% [2]. They are mostly attributed to atherosclerosis but could be congenital and could be the sequela of vasculitic coronary disorders [3,4].

We herein report clinical and angiographic features of ectatic coronary arteries in three patients, assuming the morphological form of CAE, one combined with coronary slow flow, one with myocardial bridge, and the other presenting with myocardial infarction, giving us an insight into diverse presentations of CAE.

## Case presentation

Case 1

A 73-year-old man with a history of hypertension, hyperlipidemia, and tobacco use presented with six months history of exertional angina. He had a normal physical examination and a left bundle branch block on a standard electrocardiogram (ECG). His transthoracic echocardiogram showed normal left ventricle function and no significant valve disease. He also underwent stress echocardiography, which was positive.

**Figure 1:**
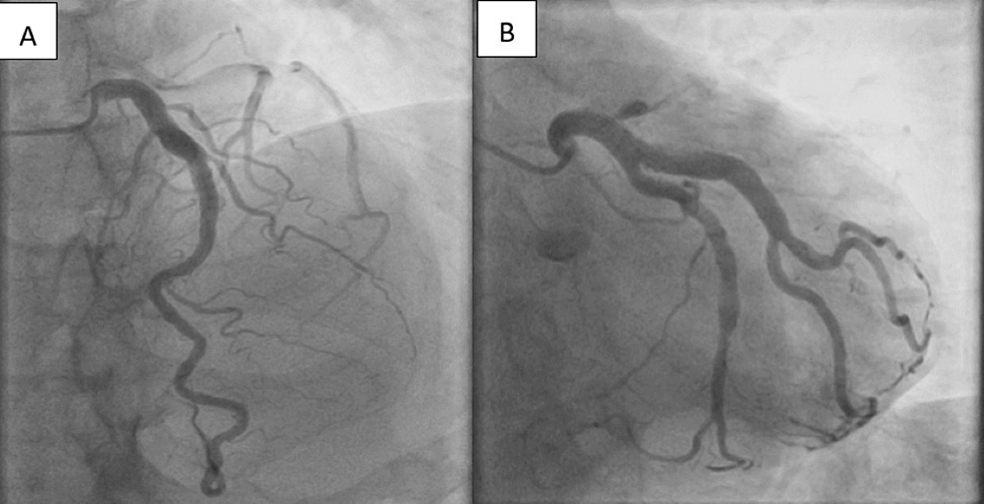
Multiple ectatic coronary segments. (A) The proximal part of the left anterior descending artery and (B) the proximal left circumflex and its obtuse marginal branch with sluggish intraluminal flow.

Coronary angiography showed ectasia of the proximal left circumflex (LCX) artery, its obtuse marginal branch, and the left anterior descending artery (LAD), in its proximal to the middle part, with turbulent flow and delayed antegrade dye filling (Figure 1).

Case 2

A 62-year-old woman with a history of hypertension presented with worsening dyspnea over the prior three months. However, she denied any chest pain or angina equivalent, syncope, or palpitations. She had a blood pressure of 130/108, a heart rate of 90 beats per minute, and a respiratory rate of 18 breaths per minute. She had no signs of heart failure at the time of examination.

Her admission ECG showed a left bundle branch block, and her transthoracic echocardiogram showed left ventricular dilation with important mechanical dyssynchrony and moderate systolic dysfunction.

**Figure 2:**
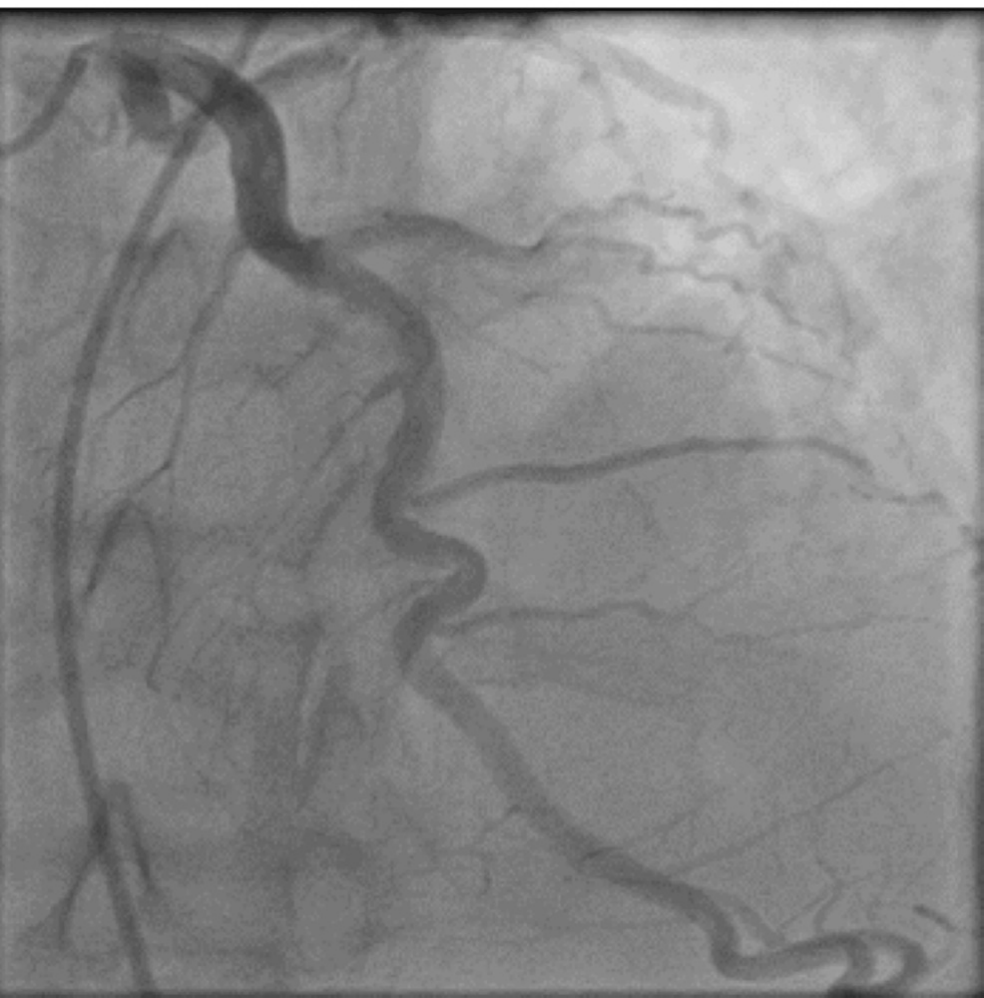
Moderately ectatic left anterior descending artery in its proximal and middle parts with a proximal swirling flow and a distal myocardial bridging.

Coronary angiography showed a moderately ectatic LAD artery with a distal delay in coronary vessel opacification. It also revealed a systolic narrowing of its distal segment with a "step down-step up" phenomenon suggestive of a tunneled segment (Figure 2).

Case 3

A 45-year-old man developed sudden onset, severe central chest pain, associated with diaphoresis and nausea. The patient had multiple cardiovascular risk factors, including hypertension and cigarette smoking.

On examination, the patient was anxious and alert, his heart rate was 100 beats per minute, his blood pressure was 140/82 mmHg, and his respiratory rate was 24 breaths per minute.

The ECG revealed a sinus rhythm, heart rate of 100 beats per minute, and symmetric T wave inversions in leads V3 through V6 with evidence of left ventricular hypertrophy. The initial serum troponin level was 5.2 ng/mL (the upper limit of the normal is 0.039 ng/mL).

**Figure 3:**
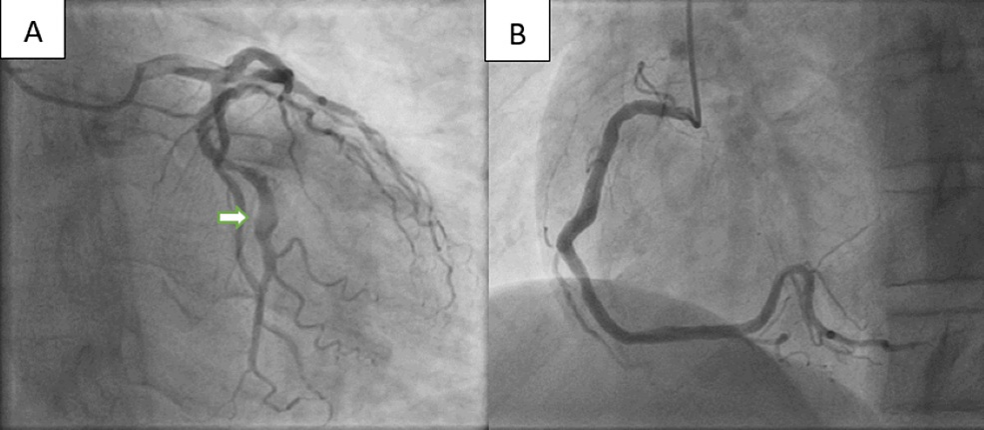
(A) Fusiform ectasia of the middle segment of left circumflex artery (white arrow). (B) Diffuse mild ectasia of the right coronary artery.

Emergency coronary angiography showed a mild ectasia of the right coronary artery (RCA) and a fusiform ectasia of the middle segment of LCX with a sluggish, swirling blood flow and no flow-limiting stenosis (Figure 3).

The patient received low molecular weight heparin and dual antiplatelet therapy. He was instructed to return for a follow-up, showing improved clinical symptoms.

## Discussion

The term "ectasia" refers to an extended widening of a tubular structure [5]. CAE is an anatomical variant and a phenotypic expression of coronary artery disease, that is defined as a coronary artery lumen dilatation with a diameter of 1.5 times the adjacent healthy coronary artery [6].

The etiology of CAE can be murky. Atherosclerosis is considered the principal etiologic cause that is responsible for greater than 50% of cases in adults [6]. CAE likely represents a form of expansive vascular remodeling secondary to atherosclerotic plaque growth [7].

Based on morphology and on the extent of ectatic involvement, Markis et al. suggested four types of CAE: type I (diffuse dilatation in more than two coronary vessels), type II (diffuse ectasia in a vessel associated with localized dilatation in the other vessel), type III (diffuse ectasia located in only one vessel), and type IV (localized or segmental involvement) [1]. Based on this morphological classification, our cases were type I, type III combined with a myocardial bridging, and type II, respectively.

Coronary angiography remains the gold standard in diagnosing CAE, providing information for their shape, size, topography, and extent, and also evidencing the presence of coexistent coronary stenoses [2]. Disturbances in blood flow filling and washout are almost constantly associated with CAE, being the direct result of inappropriate coronary dilatation, and are inherent to the severity of CAE. Signs of stagnant flow include delayed antegrade contrast filling, segmental backflow, and stasis in the ectatic coronary segment [6].

The clinical presentation of coronary aneurysms varies from asymptomatic to atypical chest pain, stable angina, and acute coronary syndromes [2]. Additionally, stress-induced ischemia due to microvascular dysfunction in dilated coronary arteries has also been documented in patients who have CAE with no obstructive coronary lesions [8].

As in our cases, one patient was asymptomatic altogether and was diagnosed with incidental non-giant coronary artery diffuse ectasia, another presented with angina and a positive stress echo test, and the other was admitted for non-ST elevation myocardial infarction.

The management of coronary ectasia is substantially challenging owing to the poorly understood mechanisms and the scarcity of data suggesting a treatment strategy. Based on the significant flow disturbances within the ectatic segments, chronic anticoagulation has been proposed as the main therapy. However, this treatment has not been prospectively tested, thus, cannot be recommended routinely, unless supported and approved by further studies [9].

## Conclusions

Although CAE has been reported and largely described, many aspects, i.e., clinical, angiographic, and therapeutic, are still unknown. CAE accompanies atherosclerotic coronary disease in the vast majority of cases and might be presented in asymptomatic patients, and also in patients describing exertional symptoms or acute coronary syndromes. The co-existence of coronary ectasia with other angiographic features such as myocardial bridge and coronary slow flow should be reported and recognized by clinicians, as a data basis for further studies and investigations.
